# Antiwrinkle and Antimelanogenesis Effects of Tyndallized *Lactobacillus acidophilus* KCCM12625P

**DOI:** 10.3390/ijms21051620

**Published:** 2020-02-27

**Authors:** Hye Yeon Lim, Deok Jeong, Sang Hee Park, Kon Kuk Shin, Yo Han Hong, Eunji Kim, Yeong-Gyeong Yu, Tae-Rahk Kim, Hun Kim, Jongsung Lee, Jae Youl Cho

**Affiliations:** 1 Department of Biocosmetics, Sungkyunkwan University, Suwon 16419, Korea; gosl177@naver.com (H.Y.L.); 84701@naver.com (S.H.P.); 2 Department of Integrative Biotechnology, Sungkyunkwan University, and Biomedical Institute for Convergence at Sunkyunkwan University (BICS), Suwon 16419, Korea; gamad86@nate.com (D.J.); shuka337@naver.com (K.K.S.); ghddygks13@naver.com (Y.H.H.); im144069@gmail.com (E.K.); 3 Elishacoy Co., Ltd., Seoul 06193, Korea; utmgm12@elishacoy.com (Y.-G.Y.); james@elishacoy.com (H.K.); 4 Centre for Research and Development, LactoMason Co., Ltd., Jinju 52840, Korea; trkim@lactomason.com

**Keywords:** *Lactobacillus* acidophilus, antiwrinkle, antimelanogenesis, MMPs, AP-1

## Abstract

UVB irradiation can induce generation of reactive oxygen species (ROS) that cause skin aging or pigmentation. *Lactobacillus acidophilus* is a well-known probiotic strain that regulates skin health through antimicrobial peptides and organic products produced by metabolism and through immune responses. In this study, we investigated the antioxidative, antiwrinkle, and antimelanogenesis effects of tyndallized *Lactobacillus acidophilus* KCCM12625P (AL). To analyze the effects of AL on UV irradiation-induced skin wrinkle formation in vitro, human keratinocytes and human dermal fibroblasts were exposed to UVB. Subsequent treatment with AL induced antiwrinkle effects by regulating wrinkle-related genes such as matrix metalloproteinases (MMPs), SIRT-1, and type 1 procollagen (COL1AL). In addition, Western blotting assays confirmed that regulation of MMPs by AL in keratinocytes was due to regulation of the AP-1 signaling pathway. Furthermore, we confirmed the ability of AL to regulate melanogenesis in B16F10 murine melanoma cells treated with α-melanocyte-stimulating hormone (*α*-MSH). In particular, AL reduced the mRNA expression of melanogenesis-related genes such as tyrosinase, TYRP-1, and TYRP-2. Finally, we used Western blotting assays to confirm that the antimelanogenesis role of AL was due to its regulation of the cyclic adenosine monophosphate (cAMP) signaling pathway. Collectively, these results indicate that AL has an antiwrinkle activity in damaged skin and can inhibit melanogenesis. Thus, AL should be considered an important substance for potential use in anti-aging drugs or cosmetics.

## 1. Introduction

Skin is consistently in contact with the external environment, protecting the body from harmful factors. The skin not only protects the body from viruses and bacteria, but also prevents moisture loss [1]. The skin aging system is composed of several processes including those of hereditary factors and environmental factors that are normally associated with sun exposure. Repeated exposure to UVB irradiation can induce generation of reactive oxygen species (ROS) to cause skin aging and pigmentation [2,3]. Moreover, the increase in ROS generated by UV irradiation not only induces cell death, but also increases expression levels of matrix metalloproteinases (MMPs) [4,5,6]. This process is characterized by formation of coarse wrinkles, thickening of skin, and dryness [7,8,9,10]. In melanocytes, ROS regulate melanogenesis [11,12], and UVB irradiation stimulates keratinocytes to induce α-melanocyte-stimulating hormone (*α*-MSH) secretion, which triggers cell signaling in response to melanocortin 1 receptor (MC1R) of melanocytes [6]. In melanogenesis, the tyrosinase protein is regulated by the MC1R signaling pathway. When mouse melanoma cells promote melanogenesis, they are activated by *α*-MSH in the order of protein kinase A (PKA), cAMP response element binding (CREB), and protein microphthalmia-associated transcription factor (MITF) [13,14,15]. Melanogenesis is the resulting physiological phenomenon of this signaling pathway [16]. If excessively increased, melanogenesis is a major factor affecting quality of life due to aesthetic dissatisfaction. 

Chronic exposure to UVB irradiation injures skin, causing premature wrinkles, dryness, thinning, and increased pigmentation [6,17,18], as well as acute inflammation such as edema and erythema [19,20,21,22]. Most of these phenomena occur due to damage to the dermis, where fibroblast cells are located. Increased exposure to UVB radiation causes increased ROS (including high ROS levels in the skin), increased expression level of MMP-1, and degradation of dermal collagen, which collectively impair the function of the skin barrier. Specifically, UVB-induced MMP-1 expression and degradation of dermal collagen result in visibly dry and aged skin [23,24]. 

Probiotics are live microorganisms that contribute to host benefits such as intestinal health [25,26]. In addition, oral treatment with probiotics has been reported to affect skin health [26]. *Lactobacillus acidophilus* is a widely-studied probiotic strain [27] that regulates the immune response through production of antimicrobial peptides and organic metabolites [28].

*Lactobacillus acidophilus* is a probiotic strain that regulates immune responses through antimicrobial peptides and organic products generated by metabolism [27,28]. Oral treatment with probiotics affects skin health [26], and recent studies have reported that *L. acidophilus* IDCC 3302 affects skin biological responses by exerting antiphotodamage, antiwrinkle, and skin moisturizing effects [29,30,31]. 

In this study, we examined the effects of heat-killed (tyndallized) *L. acidophilus* KCCM12625P (AL) on the skin’s biological responses to UVB irradiation, such as AL’s antioxidant, antiwrinkle, and antimelanogenesis effects, using human keratinocytes, human dermal fibroblast (HDF) cells, and B16F10 murine melanoma cells. In particular, we found that AL regulates ROS, MMPs, and the AP-1 signaling pathway in ultraviolet-irradiated keratinocytes and HDF cells. Additionally, we used B16F10 melanoma cells to demonstrate for the first time that the antimelanogenesis effects of AL occur through regulation of the cyclic adenosine monophosphate (cAMP) signaling pathway.

## 2. Results

### 2.1. In Vitro Antioxidant Effects of AL in Skin Cells

**Figure 1:**
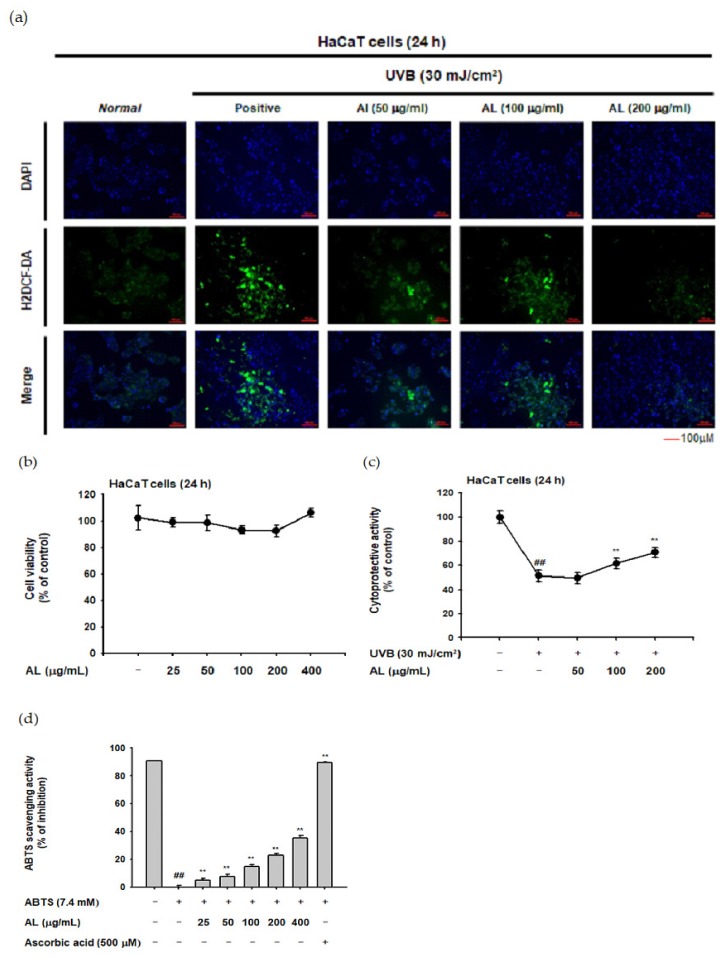
In vitro skin antioxidant effects of tyndallized *Lactobacillus acidophilus* (AL). (**a**) Primary human keratinocyte (HaCaT) cells were subjected to ultraviolet-B (UVB) irradiation (30 mJ/cm^2^) in the absence or presence of AL (50–200 µg/mL), and the resulting reactive oxygen species (ROS) levels were determined with a H2DCFDA staining assay. (**b**) Cell viability of HaCaT cells treated with the indicated dose of AL (50–200 µg/mL) for 24 h was measured using the tetrazolium colorimetric (MTT) assay. (**c**) HaCaT cells were subjected to UVB irradiation (30 mJ/cm^2^) and treated with the indicated dose of AL (50–200 µg/mL) for 24 h. The cytoprotective effects of AL were measured using the MTT assay. (**d**) The ABTS radical scavenging activity of AL at the indicated concentration (25–400 µg/mL) was measured. +: indicate treatment, −: indicate non-treatment. For all applicable experiments, statistical significance was evaluated using the Mann–Whitney *U* test. ## *p* < 0.01 compared with the normal group, ** *p* < 0.01 compared with the control group.

To investigate whether AL reduces ROS generation, the H2DCFDA-staining assay was used. ROS generation was induced by UVB irradiation (30 mJ/cm^2^) of HaCaT cells, and AL reduced the ROS levels in a dose-dependent manner (Figure 1a). In the MTT assay, AL did not show cell cytotoxicity in the concentration range of 25–400 µg/mL AL (Figure 1b). The cell viability of HaCaT cells was decreased by UVB irradiation (30 mJ/cm^2^) and recovered by AL, implying a cytoprotective effect against cell death caused by oxidative stress (Figure 1c). The antioxidant effect of AL was further investigated in vitro using a radical-scavenging activity assay. 2,2’-azino-bis (3-ethylbenzothiazoline-6-sulfonic acid (ABTS) was incubated with either AL at a concentration of 25-400 µg/mL or ascorbic acid (500 µM) as a positive control for 20 min. AL reduced the ABTS radical level in a density-dependent manner (Figure 1d). Taken together, these data strongly suggest that AL has antioxidant effects. 

### 2.2. Antiwrinkle Effects of AL through Activation of the AP-1 Signaling Pathway in HaCaT Cells

**Figure 2:**
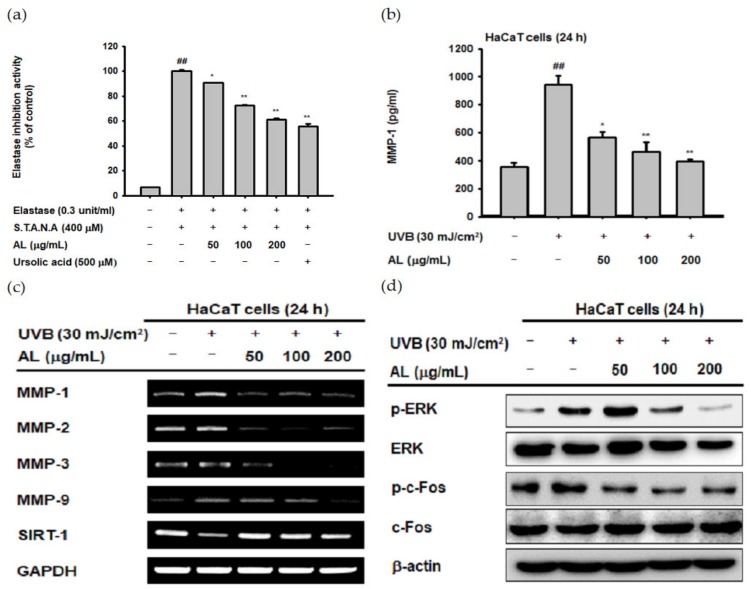
Antiwrinkle effects of AL due to activation of the activator protein 1 (AP-1) signaling pathway in HaCaT cells. (**a**) Following pretreatment with AL (50–200 µg/mL), HaCaT cells were incubated with elastase (0.3 unit/mL) and STANA (400 µM), and the elastase inhibition was measured. (**b**) The expression of matrix metalloproteinases (MMP-1) in HaCaT cells treated with AL (50–200 µg/mL) following UVB irradiation was measured using ELISA. (**c**) The mRNA expression of MMPs and SIRT-1 in HaCaT cells treated with the indicated concentrations of AL (50–200 µg/mL) were determined using the RT-PCR analysis. (**d**) ERK and c-Fos expression levels in cells treated with AL (50–200 µg/mL) were analyzed using Western blotting. +: indicate treatment, −: indicate non-treatment. For all applicable experiments, statistical significance was evaluated using the Mann–Whitney *U* test. ## *p* < 0.01 compared with the baseline group, and * *p* < 0.05 and ** *p* < 0.01 compared with the positive control group.

ROS induced by UVB irradiation contributes to intrinsic aging such as photoaging. In particular, ROS induce wrinkles by inducing degradation of the extracellular matrix (ECM) through induction of MMPs and elastase enzymes in keratinocytes and fibroblasts [32,33,34]. To confirm the antiwrinkle effect of AL, we measured its elastase inhibition activity [35]. AL inhibited elastase activity in a dose-dependent manner (Figure 2a). MMP-1 expression was determined using ELISA, and the amount of MMP-1 induced by UVB was inhibited by AL (Figure 2b). These data indicate that AL exerts antiwrinkle effects through inhibition of MMP-1 and elastase in keratinocytes with UVB-induced oxidative stress.

UVB irradiation in human skin cells induces the expression level of other MMPs in addition to MMP-1 [36]. To clarify the antiwrinkle effects of AL regarding UVB-induced MMP expression, we determined the mRNA Fseveral MMPs, as well as sirtuin 1 (SIRT-1) under UVB-induced oxidative stress conditions using RT-PCR. AL suppressed the expression of MMP-1, -2, -3, and -9 in UVB-induced HaCaT cells in a dose-dependent manner. Additionally, the expression of SIRT-1, which has notable functions in cell survival and inflammatory processes [37,38,39], was increased to approximately its baseline level by AL (Figure 2c). ROS, which are secondary messengers to activate mitogen-activated protein kinase (MPAK), promote the expression of MMPs due to induction of the AP-1 signaling pathway [36]. Therefore, we conducted Western blotting to determine whether reduction of ROS due to the antioxidant effect of AL contributes to inhibition of AP-1 signal activity. AL significantly inhibited the AP-1 pathway by inhibiting phosphorylation of ERK and c-Fos in UVB-irradiated keratinocytes (Figure 2d). These data show that the ROS-scavenging effect of AL on the UVB-induced oxidative stress response leads to anti-aging effects in keratinocytes.

### 2.3. Antiwrinkle Effects of AL in Human Dermal Fibroblast Cells 

**Figure 3:**
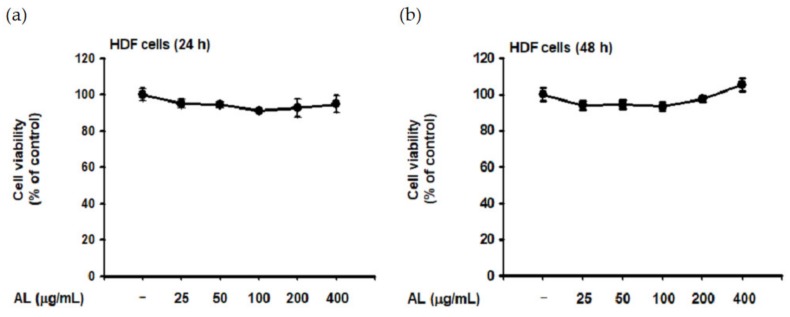

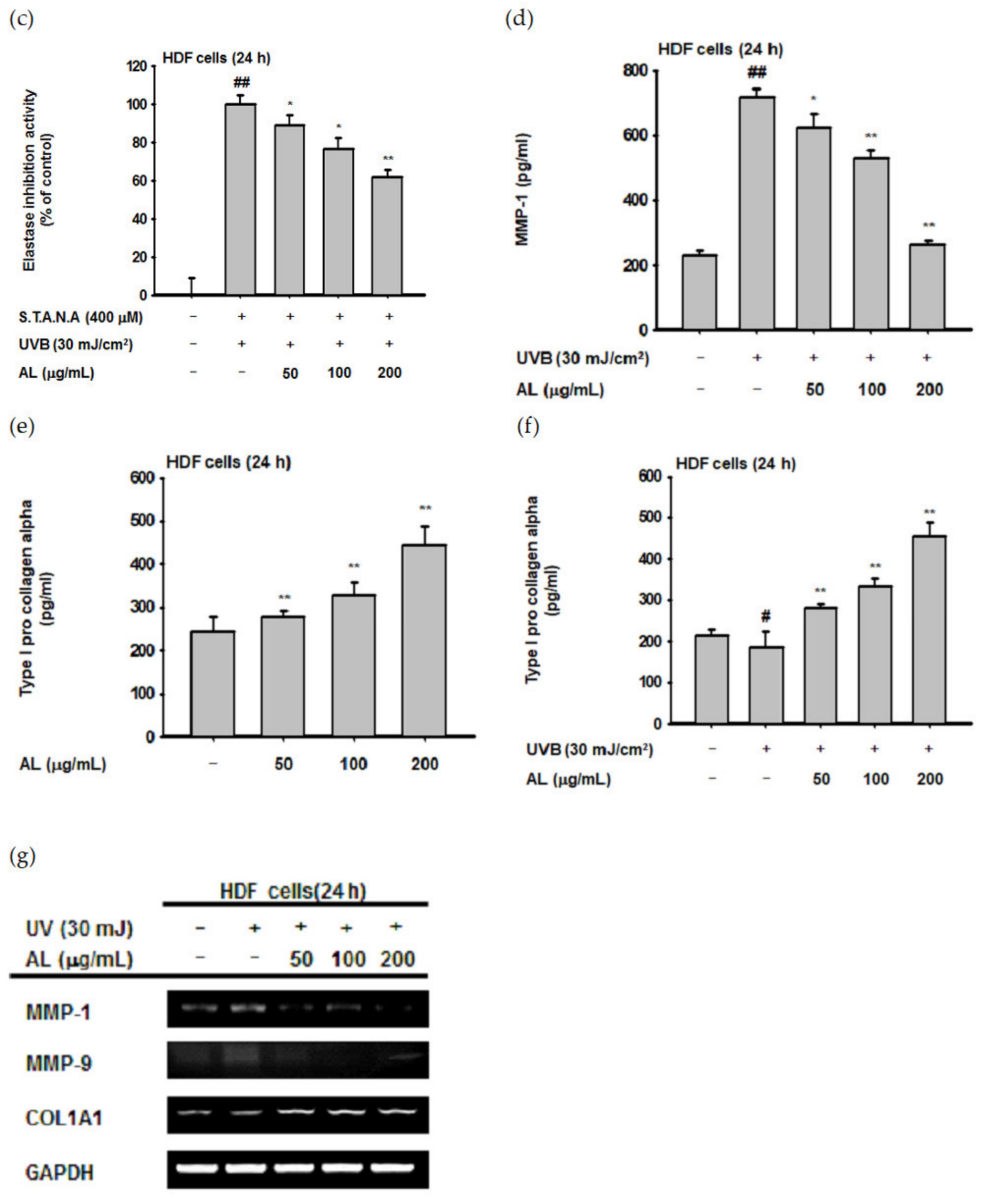
Antiwrinkle effects of AL in human dermal fibroblast cells. (**a** and **b**) Viability of human dermal fibroblast (HDF) cells treated with the indicated dose of AL (50–200 µg/mL) for 24 (**a**) or 48 h (**b**) was measured using the MTT assay. (**c**) Elastase inhibition activity of AL (50–200 µg/mL) in UVB-irradiated HDF cells was measured using STANA (400 µM). (**d**) MMP-1 expression was measured using ELISA in human dermal fibroblast cells treated with AL (50–200 µg/mL) following UVB irradiation. (**e** and **f**) Type 1 procollagen alpha expression was measured using ELISA in human dermal fibroblast cells treated with AL (50–200 µg/mL) without (**e**) and with (**f**) UVB irradiation. (**g**) The mRNA expression of MMP-1, MMP-9, and COL1A1 in HDF cells treated with AL (50–200 µg/mL) were determined using RT-PCR. +: indicate treatment, −: indicate non-treatment. For all applicable experiments, statistical significance was evaluated using the Mann–Whitney *U* test. # *p* < 0.05 and ## *p* < 0.01 compared with the normal group, and * *p* < 0.05 and ** *p* < 0.01 compared with the control group.

Though UVB is absorbed in the epidermis and has little effect on the dermis [40], such irradiation induces ROS generation of HDF cells in vitro [41,42]. Since dermal fibroblasts are the primary cells secreting MMPs and elastase [32,36], we evaluated the biological effect of UVB-induced oxidative response in HDF cells. First, the cytotoxicity of AL in HDF cells was examined by treating cells with increasing doses of AL for 24 or 48 h. Cell viability was then determined using the MTT assay, and AL had no appreciable cytotoxicity in HDF cells (Figure 3a,b). Since AL modulates elastase activity (Figure 2a), we performed an in vitro evaluation of the effects of AL in HDF following UVB irradiation. AL inhibited both elastase activity (Figure 3c) and MMP-1 expression (Figure 3d) in HDF cells after UVB-induced oxidative stress. Taken together, these data indicate that AL has antiwrinkle effects in HDFs and keratinocytes under UVB-induced oxidative stress.

Type 1 procollagen, which modulates the structure of skin tissue, is one of the major markers of wrinkle development in the skin and is degraded by MMP-1 [43]. Interestingly, the type 1 pro-collagen alpha level in HDF was significantly increased by AL with or without UVB irradiation (Figure 3e,f). This indicates that AL regulates baseline MMP expression and induces collagen production in HDF cells (Figure 3e). Finally, we clarified the effects of AL on expression of antiwrinkle-related genes using PCR analysis. The mRNA expression of MMP-1 and MMP-9 were inhibited by AL under UVB-irradiation conditions. Conversely, COL1A1 expression was increased by AL in HDF cells (Figure 3g). Collectively, these data indicate that AL inhibits elastase and MMP-1 and induces type 1 pro-collagen to exert antiwrinkle effects.

### 2.4. Antimelanogenesis Effects of AL in B16F10 Cells

**Figure 4:**
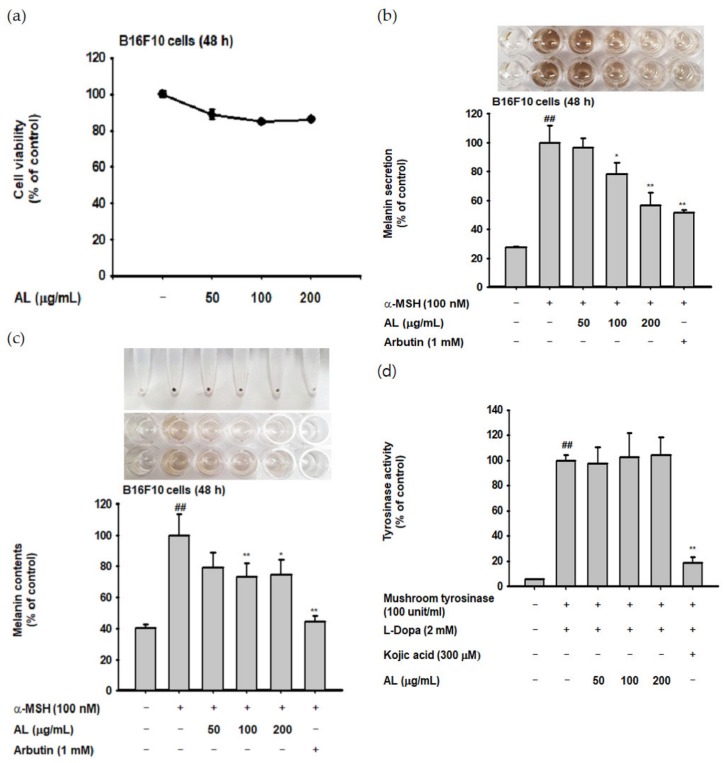

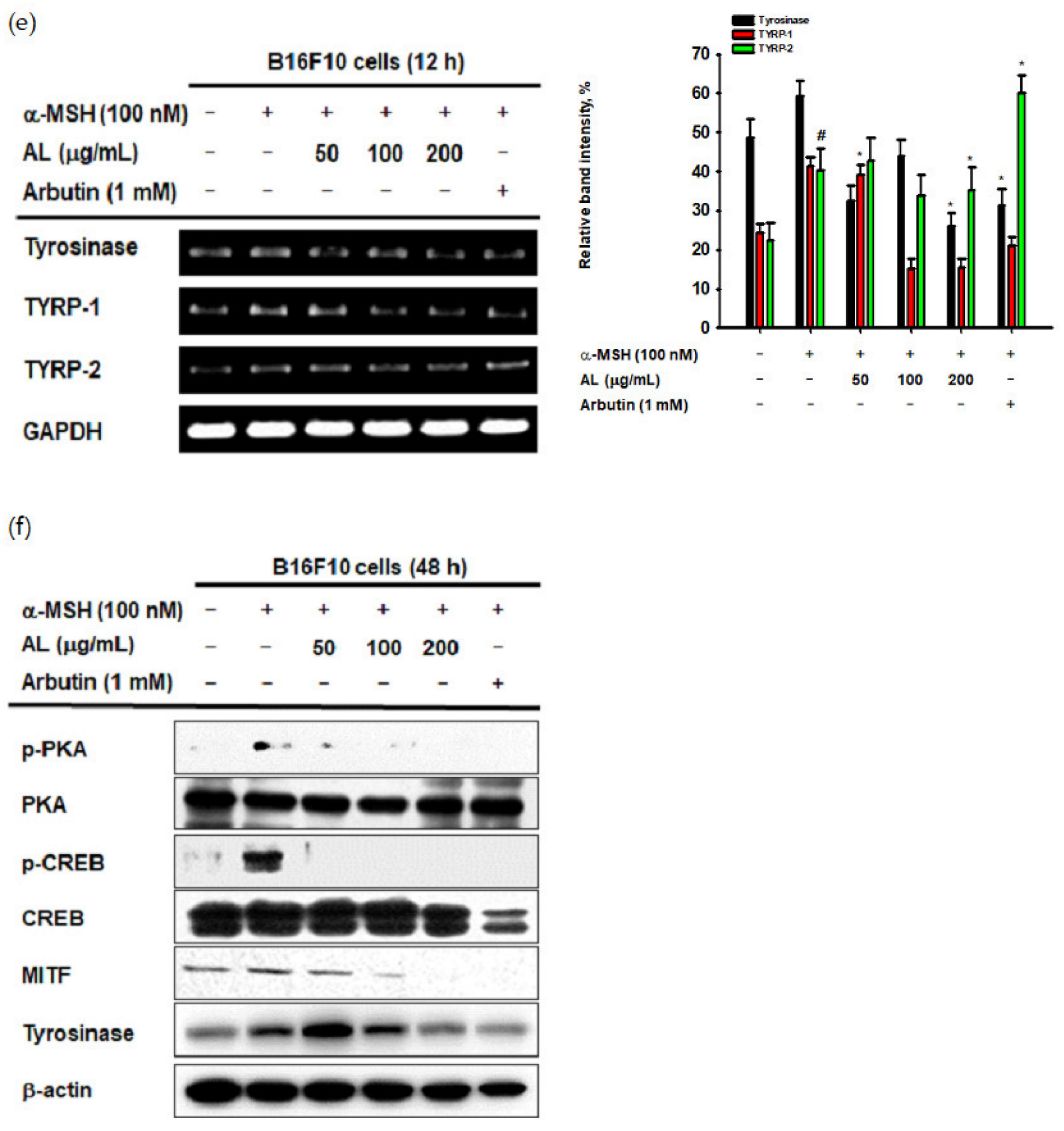
Antimelanogenesis effects of AL in B16F10 cells. (**a**) The viability of murine melanoma (B16F10) cells treated with the indicated dose of AL (50–200 µg/mL) for 48 h was measured using the MTT assay. B16F10 cells were treated with AL (50–200 µg/mL) or arbutin (1 mM) for 48 h, and melanin secretion and intracellular melanin were measured at 475 (**b**) and 405 nm (**c**), respectively. (**d**) Tyrosinase activity was measured in response to the indicated dose of AL (50–200 µg/mL) or kojic acid (300 mM). (**e**) The mRNA expression of tyrosinase, TYRP1, and TYRP-2 in B16F10 cells treated with AL (50–200 µg/mL) or arbutin (1 mM) were determined using PCR analysis. (**f**) Protein expression levels of various cyclic adenosine monophosphate (cAMP) signaling pathway proteins in response to AL (50–200 µg/mL) or arbutin (1 mM) were determined using Western blotting. +: indicate treatment, -: indicate non-treatment. For all applicable experiments, statistical significance was evaluated using the Mann–Whitney *U* test. # *p* < 0.05 and ## *p* < 0.01 compared with the normal group, * *p* < 0.05 compared with the control group, ** *p* < 0.01 compared with the control group.

UVB irradiation induces melanogenesis through physiological responses in keratinocytes. We explored the effects of AL on the melanogenesis response induced by UVB-irradiation in B16F10 cells. MTT assay confirmed that AL had no cytotoxicity in B16F10 cells at doses of 50–400 µg/mL for 48 h (Figure 4a). To investigate the effects of AL on melanogenesis, B16F10 cells were treated with AL and *α*-MSH, a melanocyte-stimulate hormone. The level of melanin secreted from the cells, as well as in the cells, was then determined. AL significantly inhibited melanin secretion (Figure 4b) and intracellular content (Figure 4c) in a dose-dependent manner. Tyrosinase is an important molecule in the melanogenesis process; therefore, we investigated the tyrosinase-inhibition activity of AL using a mushroom tyrosinase and L-3,4- dihydroxyphenylalanine (L-DOPA) assay, as well as PCR analysis. Mushroom tyrosinase activity was not inhibited by AL (Figure 4d), and AL reduced the mRNA expression of tyrosinase and TYRP-1 but not TYRP-2 (Figure 4e). These results indicate that AL does not directly affect tyrosinase activity. To clarify the molecular mechanism of AL, we examined whether it regulated the cAMP signaling pathway. In B16F10 murine melanoma cells stimulated with the melanocyte hormone *α*-MSH, AL inhibited phosphorylation of PKA and CREB and downregulated protein expression of tyrosinase and MITF (Figure 4f). These results strongly indicate that AL has antimelanogenesis effects through regulation of the cAMP signaling pathway.

## 3. Discussion

In this study, we explored the skin health benefits of tyndallized *L. acidophilus* (KCCM 12625P) in skin cells exposed to UVB irradiation to induce cell death and stimulate the photoaging process [44,45]. HaCaT cells treated with UVB irradiation showed an increase in ROS. Administration of AL inhibited ROS production (Figure 1a), and MTT assay confirmed that AL was not cytotoxic at the concentrations used (Figure 1c). In addition, treatment with AL prevented cell death by UVB, indicating that AL protects the skin and body from UVB irradiation (Figure 1b). The antioxidant effects of AL were found through ABTS radical-scavenging assays [46,47,48], which again demonstrates that AL exerts antioxidant effects (Figure 1d).

ROS induced by UV irradiation not only triggers cell death, but also increases matrix metalloproteinase (MMP) expression [4,5,6]. In addition, degradation of elastin induces wrinkles, and inhibitors of elastase activity can be used to prevent signs of aging [49,50]. We further evaluated the antiwrinkle effects of AL using keratinocytes and human dermal fibroblasts. Interestingly, AL increased the mRNA expression of SIRT-1 (Figure 2c), which has a notable function in cell survival and inflammatory processes [37,38,39]. Based on our results, it is assumed that AL protects cells through antioxidative effects, inhibits MMPs by regulating AP-1 signaling, and suppresses wrinkles by inhibiting elastase activity in keratinocytes.

Formation of skin wrinkles is a phenotype of the aging process [51,52], which is affected by several factors including genetic changes, hormonal alteration, UVB irradiation, and exposure to inflammation- or oxidation-inducing agents [1,12,38]. In terms of cellular and molecular causes of wrinkle formation, a decrease of extracellular matrix proteins such as collagen in dermal fibroblasts is known as a major cause [53]. Moreover, upregulation of the activity of matrix metalloproteinases to degrade collagen is associated with loss of extracellular matrix proteins in the skin [53]. Thus, we determined the effects of AL on extracellular matrix protein levels in HDF cells. AL reduced the mRNA expression of MMP-1 and MMP-9, important skin-related genes regulated during aging [24,54], triggered by UVB irradiation and also increased the expression of procollagen (eg., COL1A1) (Figure 3e). Therefore, these data show that AL exhibits an antiwrinkle effect by regulating MMPs and elastase in HDF cells, as well as keratinocytes. In addition, AL protects against skin barrier damage caused by UVB by inducing type I pro collagen alpha.

Excessive UVB irradiation causes melanin formation, which presents as pigmentation of the skin. In B16F10 cells stimulated with *α*-MSH, which activates various transcription factors and enzymes to induce melanin production, the mRNA expression of tyrosinase and TYRP-1 was decreased by AL (Figure 4e). To clarify the mode of action of AL in melanogenesis, we confirmed its effects on cell signaling mechanisms using Western blotting. Interestingly, AL regulated the protein levels of cAMP signaling pathway members such as tyrosinase, MITF, CREB, and PKA (Figure 4f). Collectively, these results demonstrate that AL can modulate melanogenesis though the cAMP pathway.

**Figure 5:**
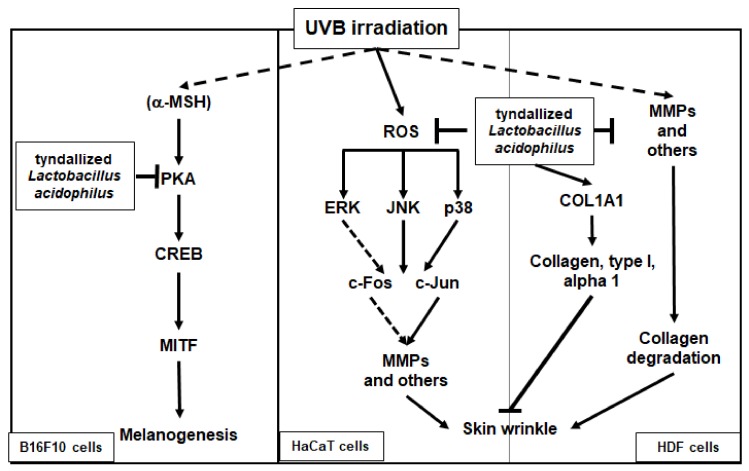
Mechanism of the antiwrinkle and antimelanogenesis effects of tyndallized *Lactobacillus acidophilus* KCCM12625P. Arrows indicate positive regulation and T- bars indicate negative regulation.

In conclusion, we demonstrated that heat-killed (tyndallized) *L. acidophilus* KCCM12625P exhibits skin-protective activities. Our results show that the skin-protective characteristics of AL are mediated through various activities. First, AL exhibited antioxidant and cell-protective activity in keratinocytes through the AP-1 signaling pathway. Second, AL inhibited wrinkle-related factors and induced cell growth in keratinocytes. Finally, AL suppressed melanin secretion and intracellular melanin content in B16F10 cells by inhibiting cAMP pathway. The skin-protective activities of AL in human keratinocytes, human dermal fibroblast cells, and B16F10 murine melanoma cells are summarized in Figure 5. Therefore, this study strongly suggests that *L. acidophilus* KCCM12625P has potential for use in anti-aging drugs or cosmetics.

## 4. Materials and Methods

### 4.1. Materials

HaCaT, HDF, and B16F10 cells were purchased from the American Type Culture Collection (Rockville, MD, USA). Heat-killed (tyndallized) *Lactobacillus acidophilus* KCCM12625P was purchased from Lactomason (Jinju, Korea). Dulbecco’s modified Eagle’s medium (DMEM), fetal bovine serum (FBS), phosphate-buffered saline (PBS), streptomycin, penicillin, and L-glutamine were purchased from Gibco (Grand Island, NY, USA). 2′,7′-dichlorofluorescein diacetate (H2DCFDA), ascorbic acid, 3-(4,5-dimethylthiazol-2-yl)-2,5-diphenyltetrazolium bromide (MTT), sodium dodecyl sulfate (SDS), 3*β*-hydroxy-12-ursen-28-ic acid (ursolic acid), N-succinyl-Ala-Ala-Ala-p-nitroanilide (STANA), phorbol 12-myristate 13-acetate (PMA), retinol (RE), LY294002, L-3,4-dihydroxyphenylalanine (L-DOPA), 5-hydroxy-2-hydroxymethyl-4H-pyranone (kojic acid), 4-hydroxyphenyl *β*-D-glucopyranoside (arbutin), and α-melanocyte stimulating hormone (α-MSH) were purchased from Sigma Chemical Co. (St. Louis, MO, USA). TRI reagent was purchased from Molecular Research Center Inc. (Cincinnati, OH, USA). MuLV reverse transcriptase was purchased from Thermo Fisher Scientific (Waltham, MA, USA). Primers specific for matrix metalloproteinases MMP-1, MMP-2, MMP-3, and MMP-9; transglutaminase-1 (TGM1); silent information regulator 1 (SIRT-1); type 1 procollagen (COL1A1); tyrosinase; TYRP-1; TYRP-2; and GAPDH used for semiquantitative reverse transcriptase polymerase chain reaction (RT-PCR) were purchased from Bioneer Inc. (Daejeon, Korea). Specific antibodies for the total- and phospho-forms of ERK, JNK, p-p38, c-Jun, c-Fos, tyrosinase, MITF, CREB, PKA, and β-actin were purchased from either Cell Signaling Technology (Beverly, MA, USA) or Santa Cruz Biotechnology (Santa Cruz, CA, USA). Enhanced chemiluminescence reagents were purchased from Ab Frontier (Seoul, Korea).

### 4.2. Cell Culture

HaCaT (primary human keratinocyte), HDF (human dermal fibroblast), and B16F10 (murine melanoma) cells were cultured in DMEM supplemented with 10% FBS and 1% streptomycin (100 mg/mL) penicillin (100 U/mL) at 37 °C in a humidified 5% CO_2_ incubator.

### 4.3. ROS Generation Assay

H2DCFDA staining assays were used to confirm reactive oxygen species (ROS) generation. HaCaT cells were treated with UVB irradiation (30 mJ/cm^2^) and further treated with AL (50–200 µg/mL) for 24 h. The cells were then incubated with H2DCFDA (10 µM) at 37 °C for 20 min and washed with PBS three times. Fluorescence was measured using a flow cytometer (EMD Millipore Co., Billerica, MA, USA)

### 4.4. MTT Assay

Cell viability assays were used to determine the cytotoxicity activity of AL. HaCaT cells, human dermal fibroblast cells, or B16F10 cells were treated with AL (25–400 µg/mL) for 24 or 48 h. To confirm the cytoprotective activity of AL, oxidative stress was stimulated using UVB irradiation (30 mJ/cm^2^), and cells were treated with AL (50–200 µg/mL) for 24 h. Absorbance was measured at 570 nM.

### 4.5. Radical-Scavenging Activity Assay

The radical-scavenging activity of AL was measured using the ABTS assay as previously reported [55]. AL (25–400 µg/mL) or ascorbic acid (100 µM) was mixed with ABTS solution (7.4 mM) in a 96-well plate. The plate was incubated for 20 min at room temperature, and the absorbance was measured at 730 nM using a SpectraMax 250 microplate reader (Molecular Devices, Sunnyvale, CA, USA).

### 4.6. Elastase Inhibition Assay

Elastase (0.3 units/mL) was incubated with either AL (50–200 µg/mL) or ursolic acid (500 µM) for 30 min in a humidified 5% CO_2_ incubator. The substrate STANA (400 µM) was then added, and the reaction was incubated for 15 min in a humidified 5% CO_2_ incubator. The absorbance of the mixtures was measured at 410 nM using a SpectraMax 250 microplate reader.

### 4.7. RT-PCR

HaCaT or HDF cells were induced with UVB irradiation (30 mJ/cm^2^) and then treated with AL (50–200 µg/mL) for 24 h. In B16F10 cells, oxidative stress was stimulated by *α*-MSH for 30 min, and the cells were treated with AL (50–200 µg/mL) for 12 h. For all cell types, RNA was extracted using TRI reagent as reported previously [56,57]. cDNA was synthesized from total RNA (1 µg) using MuLV reverse transcriptase according to the manufacturer’s instructions. Primers for RT-PCR were listed in Table 1.

### 4.8. Western Blotting

HaCaT cells were treated with UVB irradiation (30 mJ/cm^2^) and AL (50–200 µg/mL) for 24 h. Western blotting was conducted as previously described [56,57]. Briefly, total cell lysates were separated using SDS-polyacrylamide gel electrophoresis and transferred to polyvinylidene fluoride membranes. Specific antibodies were used to detect the total and phosphorylated forms of the target proteins, and protein bands were visualized using enhanced chemiluminescence reagents.

### 4.9. ELISA

HDF cells (1 × 10^5^ cells/well) were seeded into 6-well plates and incubated for 24 h. The culture media was changed to fresh media supplemented with AL (50–200 µg/mL) and incubated for 24 h. The media was collected from each well, and the levels of MMP-1 and type 1 procollagen alpha were measured using ELISA assay kits (R&D Systems, Inc., Minneapolis, MN, USA) according to the manufacturer’s instructions.

### 4.10. Tyrosinase Activity Assay

Mushroom tyrosinase (100 unit/mL) was incubated with AL (50–200 µg/mL) or kojic acid (300 µM) for 30 min in a humidified 5% CO_2_ incubator. The reactions were then treated with L-DOPA (40 g/mL) for 5 min. Tyrosinase activity was determined from the absorbance of the mixture at 475 nm, which was measured with a SpectraMax 250 microplate reader.

### 4.11. Melanin Generation Assay

B16F10 cells (0.5 × 10^5^ cells/well) were seeded into 12-well plates and incubated for 24 h. The culture media was changed to fresh media supplemented with either AL (50–200 µg/mL) or arbutin (1 mM). Melanin production was then stimulated with α-MSH (100 nM) for 48 h. To determine the amount of melanin secreted into the culture media, the absorbance of the culture media was measured at 475 nm using a SpectraMax 250 microplate reader. To determine the intracellular melanin content, harvested cells were lysed using a lysis buffer (50 mM Tris HCl pH 7.5, 20 mM glycerophosphate pH 7.5, 120 mM NaCl, and 2 % NP-40) and centrifuged at 10,000 × *g* for 3 min. The concentrated cell pellets were resuspended in 10% DMSO in 1 N NaOH and incubated at 60 °C for 10 min. The absorbance at 405 nm was measured using a SpectraMax 250 microplate reader.

### 4.12. Statistical Analysis

All data are presented as mean ± standard deviation (SD) of at least three independent experiments. The experimental and control groups were compared using the Mann–Whitney *U* test, and a *p*-value < 0.05 was considered statistically significant. SPSS software (SPSS Inc., Chicago, IL, USA) was used for all statistical analyses.

## Figures and Tables

**Table 1:** Sequences of primers (human) used in semiquantitative RT-PCR.

Gene	Direction	Sequence (5’ to 3’)
*MMP-1*	Forward	TCTGACGTTGATCCCAGAGAGCAG
	Reverse	CAGGGTGACACCAGTGACTGCAC
*MMP-2*	Forward	AAAACGGACAAAGAGTTGGCA
	Reverse	CTGGGGCAGTCCAAAGAACT
*MMP-3*	Forward	TGTTAGGAGAAAGGACAGTGGTC
	Reverse	CGTCACCTCCAATCCAAGGAA
*MMP-9*	Forward	GCCACTTGTCGGCGATAAGG
	Reverse	TCGCGGGAAGAATAGGATTGG
*SIRT-1*	Forward	TCGCAACTATACCCAGAACATAGACA
	Reverse	CTGTTGCAAAGGAACCATGACA
*COL1A1*	Forward	AGGGCCAAGACGAAGACATC
	Reverse	AGATCACGTCATCGCACAACA
*Tyrosinase*	Forward	GTCCACTCACAGGGATAGCAG
	Reverse	AGAGTCTCTGTTATGGCCGA
*TYRP-1*	Forward	ATGGAACGGGAGGACAAACC
	Reverse	TCCTGACCTGGCCATTGAAC
*TYRP-2*	Forward	CAGTTTCCCCGAGTCTGCAT
	Reverse	GTCTAAGGCGCCCAAGAACT
*GAPDH*	Forward	ACCACAGTCCATGCCATCAC
	Reverse	CCACCACCCTGTTGCTGTAG

## Abbreviations

UVR	Ultraviolet radiation
AP-1	Activator protein 1
ROS	Reactive oxygen species
PCR	Polymerase chain reaction
MMP	Matrix metalloproteinases
AL	Tyndallized Lactobacillus acidophilus
IL	Interleukin
KGF	Keratin growth factor
PKA	Protein kinase A
*α*-MSH	*α*-Melanocyte-stimulating hormone
CREB	cAMP response element binding
MITF	Microphthalmia-associated transcription factor
cAMP	Cyclic adenosine monophosphate
